# The Life and Contributions of Dr. Abraham Colles: A Pioneer in Orthopaedics

**DOI:** 10.7759/cureus.71331

**Published:** 2024-10-12

**Authors:** Sagar Gurnani, Mukesh O Phalak, Archit Gupta

**Affiliations:** 1 Orthopaedics, Dr. D. Y. Patil Medical College, Hospital and Research Centre, Dr. D. Y. Patil Vidyapeeth (Deemed to be University), Pune, IND

**Keywords:** colles' fracture, congenital syphilis treatment, history of medicine, operative orthopedic surgery, surgical procedures

## Abstract

Abraham Colles (1773-1843) was a pioneering Irish surgeon whose contributions significantly advanced the fields of anatomy and surgery. Best known for describing the "Colles' fracture" of the distal radius, Colles' meticulous clinical observations and innovative treatment methods remain influential in modern orthopaedic practice. Born in Kilkenny, Ireland, Colles pursued medical education at Trinity College Dublin and the University of Edinburgh before training in London under the renowned surgeon Astley Cooper. Upon his return to Dublin, Colles was appointed resident surgeon at Steevens' Hospital, where he spent the majority of his career. Beyond his work on fractures, Colles made notable contributions to the understanding and treatment of syphilis, leading to the formulation of "Colles' law," and he introduced new surgical techniques for treating vascular conditions, including aneurysms. A dedicated educator, Colles played a central role in shaping Irish surgical practice through his teaching and leadership at the Royal College of Surgeons in Ireland. His legacy endures not only through his clinical innovations but also through his impact on medical education and public health in Ireland and beyond.

## Introduction and background

**Figure 1:**
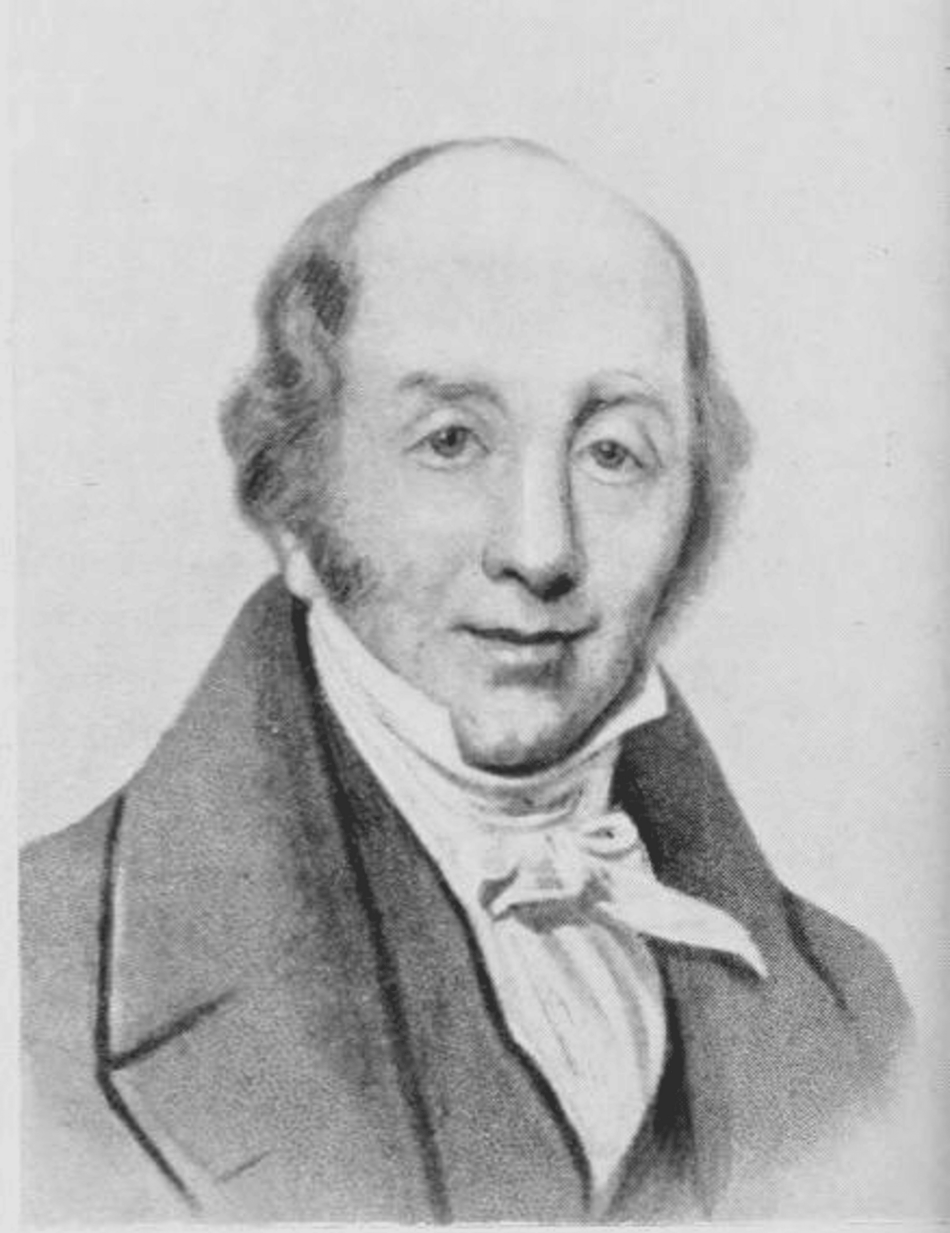
Abraham Colles This photograph is available in the public domain and is free of any known copyrights.

Dr. Abraham Colles, an eminent figure in the field of orthopaedic surgery, is best remembered for his foundational contributions to the understanding and treatment of bone fractures. Born in Ireland in the late 18th century, Colles's work laid the groundwork for modern orthopaedic practices, particularly his studies on the fracture of the distal radius, which continue to bear his name [1]. This article explores Colles's life, his major contributions to orthopaedics, and his lasting legacy in medicine (Figure 1).

## Review

Early life and education

Abraham Colles was born in 1773 in a small village in County Westmeath, Ireland. He hailed from a family with a strong educational background, which fostered his early interest in the sciences. Colles pursued his medical education at the prestigious Royal College of Surgeons in Ireland, where he demonstrated a keen intellect and a passion for surgery [2]. His education coincided with a period of significant advancements in medical science, providing him with a robust foundation to build upon.

Medical career

Colles began his medical career in the late 18th century, a time when the field of surgery was still developing. Initially, he practised general surgery, but his interests soon gravitated toward orthopaedics. His deepening fascination with bone fractures led him to focus on their diagnosis and treatment [3].

In 1803, Colles became a member of the Royal College of Surgeons in Ireland, which marked a significant milestone in his career. He went on to serve as a surgeon at several hospitals, including the Meath Hospital in Dublin. Colles’s commitment to surgical practice and education was evident as he began to teach anatomy and surgery, imparting his knowledge to a new generation of surgeons.

Major contributions

Colles Fracture

Colles’s most notable contribution to orthopaedics is his detailed description of the fracture of the distal radius, now known as a Colles fracture. In his seminal work published in 1814, titled *The Anatomy of the Bones*, Colles described the characteristics of this specific fracture, which typically occurs just above the wrist due to a fall onto an outstretched hand [4]. His meticulous observations included the angulation of the wrist and the displacement of the distal fragment, which was revolutionary for its time.

Prior to Colles, fractures were often misunderstood, and their treatment lacked the precision we recognise today. His work helped standardise the diagnosis and management of wrist fractures, greatly improving patient outcomes. The Colles fracture remains a critical concept in orthopaedic education, emphasising the importance of accurate diagnosis and appropriate treatment.

Advancements in Orthopaedic Techniques

Beyond his descriptions of specific fractures, Colles was instrumental in advancing surgical techniques. He championed the use of splints for immobilisation, recognising their significance in fracture management. His methods emphasised the need for proper alignment and stabilisation of broken bones, which laid the groundwork for modern orthopaedic practices [5].

Colles also explored various techniques for surgical intervention, including the use of bone-setting practices prevalent at the time. He critiqued these methods, advocating for a more scientific approach to surgery based on anatomical understanding. His focus on evidence-based practices was ahead of his time and highlighted the transition from traditional to modern surgical techniques.

Education and Influence

Colles’s impact extended beyond his surgical innovations. He was a passionate educator, teaching both medical students and practising surgeons. His lectures were renowned for their clarity and depth, making complex concepts accessible to learners. Colles’s commitment to education helped cultivate a generation of surgeons who would carry forward his principles of patient care and surgical excellence [6].

His influence reached far beyond Ireland; Colles was recognised internationally, and his teachings were disseminated through various medical journals and conferences. His ability to bridge the gap between theory and practice inspired many to pursue orthopaedics as a speciality.

Later Life and Legacy

As Colles aged, he continued to contribute to the field through research and teaching. He maintained an active surgical practice well into his later years, demonstrating a lifelong commitment to patient care. His peers recognised his contributions, and he became a respected figure in the medical community [7].

Dr. Abraham Colles passed away in 1843, but his legacy endures in the field of orthopaedics. The term "Colles fracture" remains a testament to his lasting impact on the understanding of wrist injuries. His emphasis on careful observation, anatomical knowledge, and patient-centred care laid the foundation for future advancements in orthopaedic surgery [8].

Influence on Modern Orthopaedics

Colles’s contributions can be seen in contemporary orthopaedic practice. The principles he established regarding fracture treatment, particularly in the context of wrist injuries, continue to guide surgeons today. His insights into the biomechanics of bone healing have influenced the development of modern fixation devices and rehabilitation protocols.

Moreover, Colles’s emphasis on education has left a profound impact on medical training [9]. The importance of teaching and mentorship in orthopaedics has become a cornerstone of the speciality, with many surgeons following in his footsteps to train the next generation of medical professionals [10].

## Conclusions

Dr. Abraham Colles’s life and work exemplify the spirit of inquiry and dedication that characterises great medical pioneers. His contributions to the understanding and treatment of bone fractures have had a lasting influence on orthopaedic surgery. By blending rigorous scientific observation with compassionate patient care, Colles set a standard that continues to inspire orthopaedic surgeons around the world.

As we reflect on his legacy, it is essential to recognise the transformative impact of his work. The principles he established not only advanced the field of orthopaedics but also enriched the practice of medicine as a whole. Colles’s enduring influence serves as a reminder of the importance of innovation, education, and dedication in the quest for improved patient outcomes in the ever-evolving field of medicine.
